# Supplementary material for: [18F]F-AraG imaging reveals association between neuroinflammation and brown- and bone marrow adipose tissue
Source: Commun Biol. 2024 Jul 1;7:793. doi: 10.1038/s42003-024-06494-x (PMC11217368; doi:10.1038/s42003-024-06494-x)
Supplement: Supplementary file 2 — Supplementary Information [file 42003_2024_6494_MOESM2_ESM.pdf]

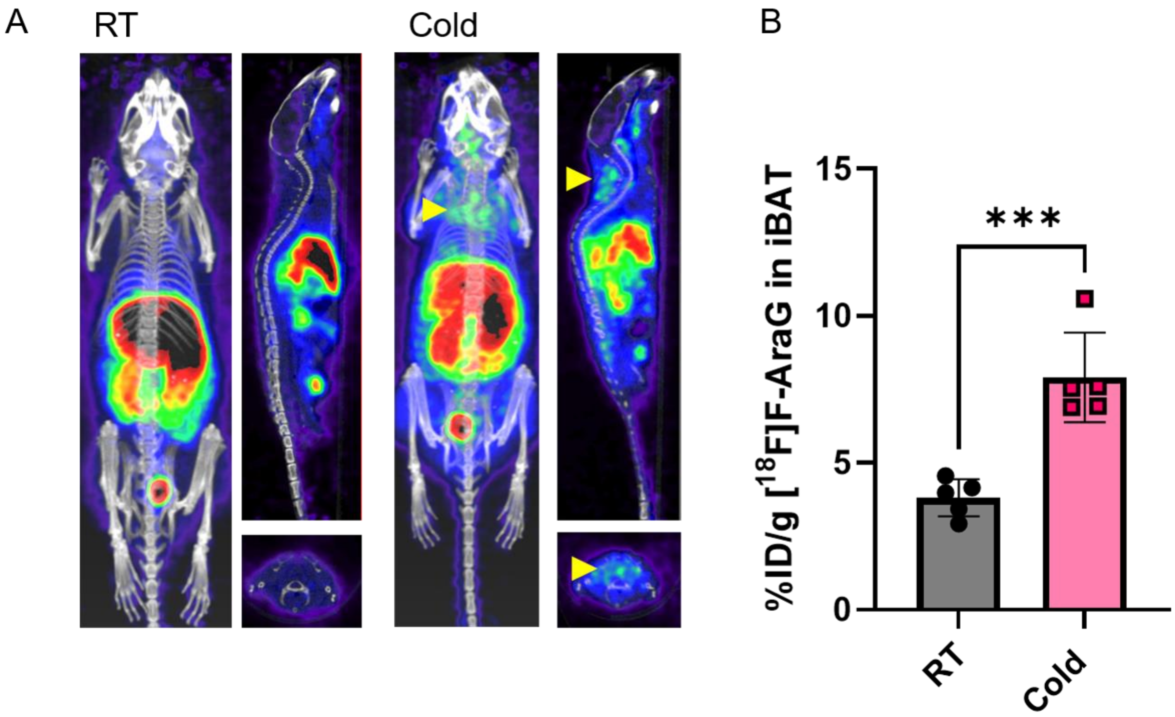

Supplementary Figure 1. [^{18}F]F-AraG accumulation in iBAT after cold exposure. Mice were kept at 4ºC for 24 hours before being imaged with [^{18}F]F-AraG. (A) Cold exposure leads to [^{18}F]F-AraG accumulation in iBAT (yellow arrow). Mice kept at room temperature (RT) do not show [^{18}F]F-AraG signal in the iBAT. (B) [^{18}F]F-AraG accumulation in iBAT of cold-exposed mice was significantly higher compared to mice maintained at room temperature.

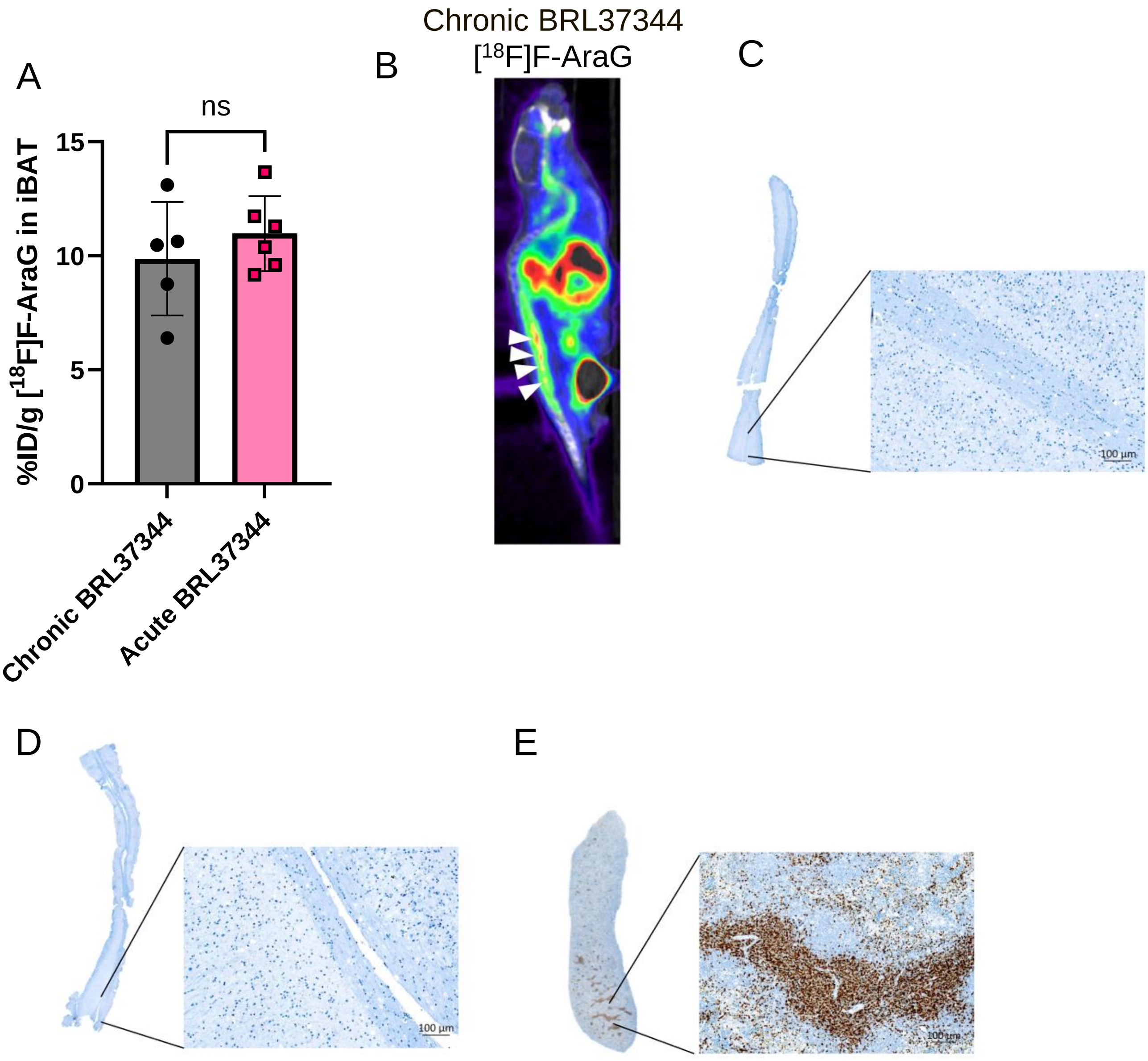

Supplementary Figure 2. [^{18}F]F-AraG accumulation in iBAT and bone marrow after chronic adrenergic stimulation. Mice were treated with β3 adrenergic receptor agonist BRL37344 (10mg/kg) injections for 4 d. (A) Accumulation of [18F]F-AraG in iBAT of acutely and chronically treated mice did not differ significantly. (B) [^{18}F]F-AraG/PET image shows the segmentary pattern of [^{18}F]F-AraG uptake in the spine region. Immunohistochemical staining for CD3 in the spinal cord from PBS-treated (C) and BRL37344-treated mice. Chronic BRL37344 treatment did not induce T cell infiltration in the spinal cord. (D) Representative image of spleen CD3 immunohistochemistry as a positive control. (E) [^{18}F]FDG-PET/CT image of glucose uptake of BRL37344-treated mice. There was no apparent [^{18}F]FDG uptake in the spinal region.

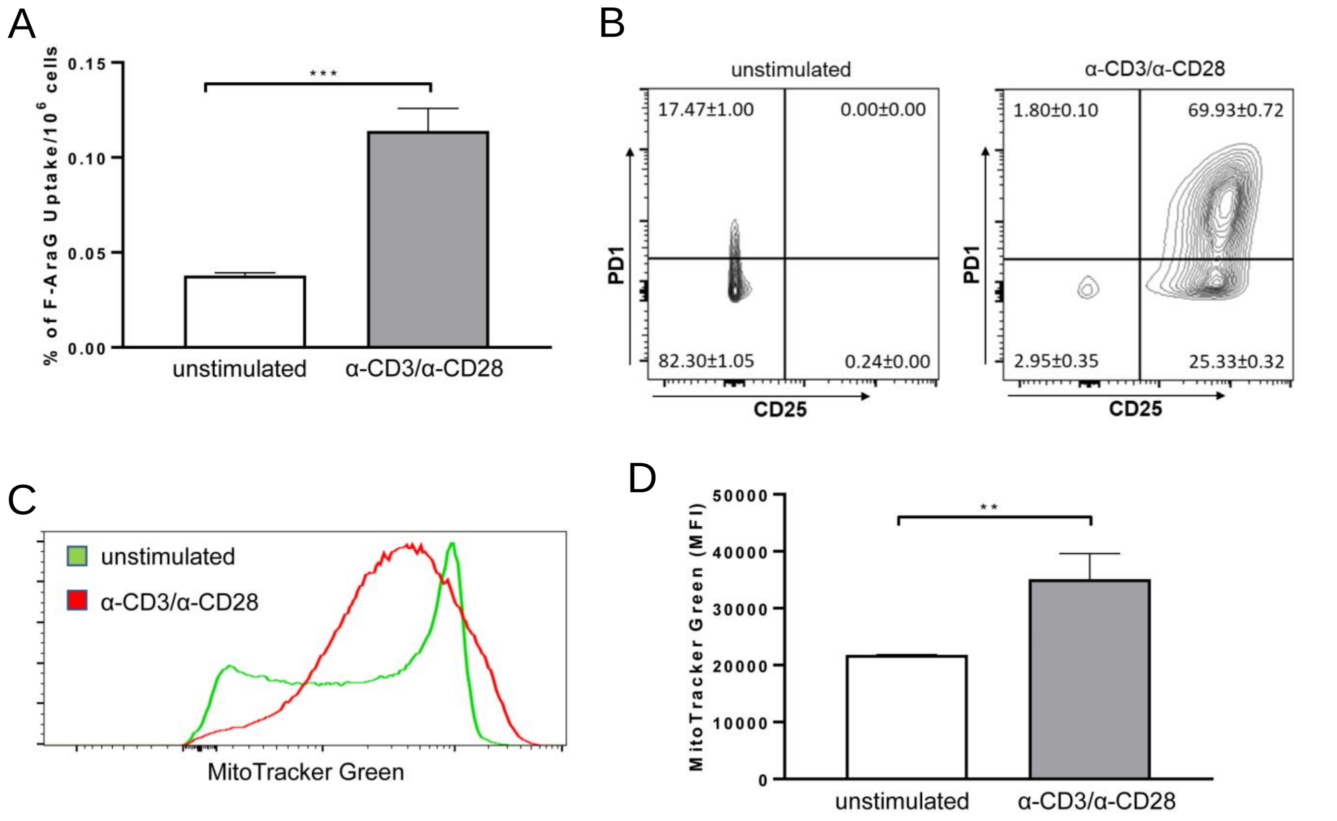

Supplementary Figure 3. Up-regulation of F-AraG uptake and mitochondrial mass in stimulated human CD8+ T cells. Human CD8+ T cells were stimulated with soluble anti-CD28 antibody for 3 days on anti-CD3 antibody-coated plates. (A) Stimulated CD8+ T cells showed higher uptake of F-AraG than the unstimulated T cells. (B) Representative flow cytometry plots showing upregulation of T cell activation markers CD25 and PD1 by anti-CD3 and anti-CD28 antibody treatment. Frequencies of cells in each gate are shown. (C & D) Cells were stained with MitoTracker Green to assess the mitochondrial mass. Representative flow cytometry histogram (C) and (D) MFI of MitoTracker Green staining are shown. Data are presented as mean ± SD (n=3).

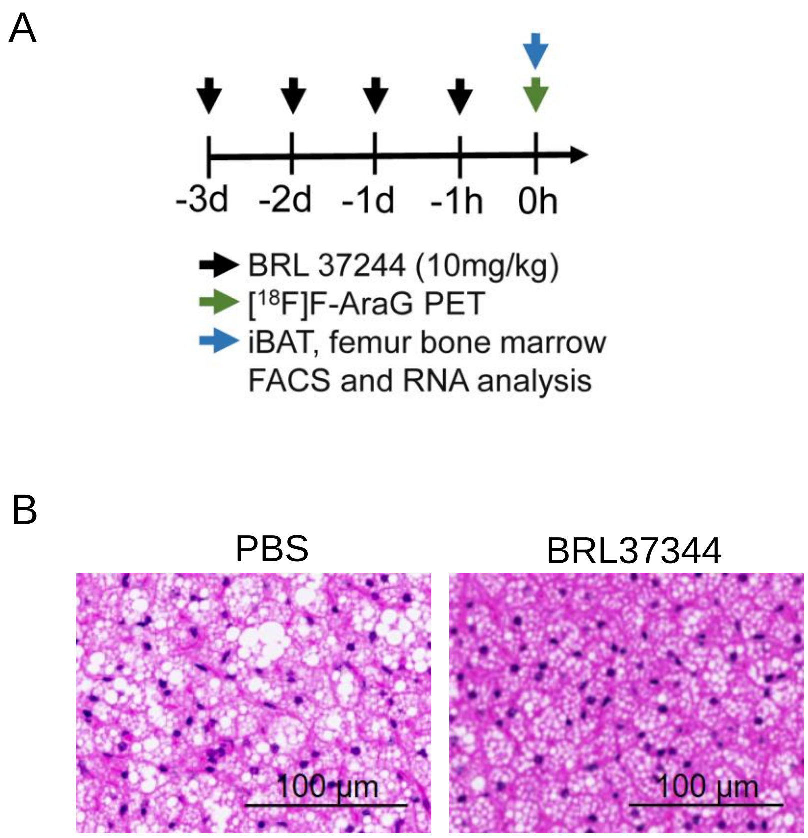

Supplementary Figure 4. (A) Diagram shows the experimental scheme. (B) Representative H&E staining images of iBAT show a high density of intracellular organelles and smaller sizes of lipid droplets and adipocytes in BRL37344-treated mice.

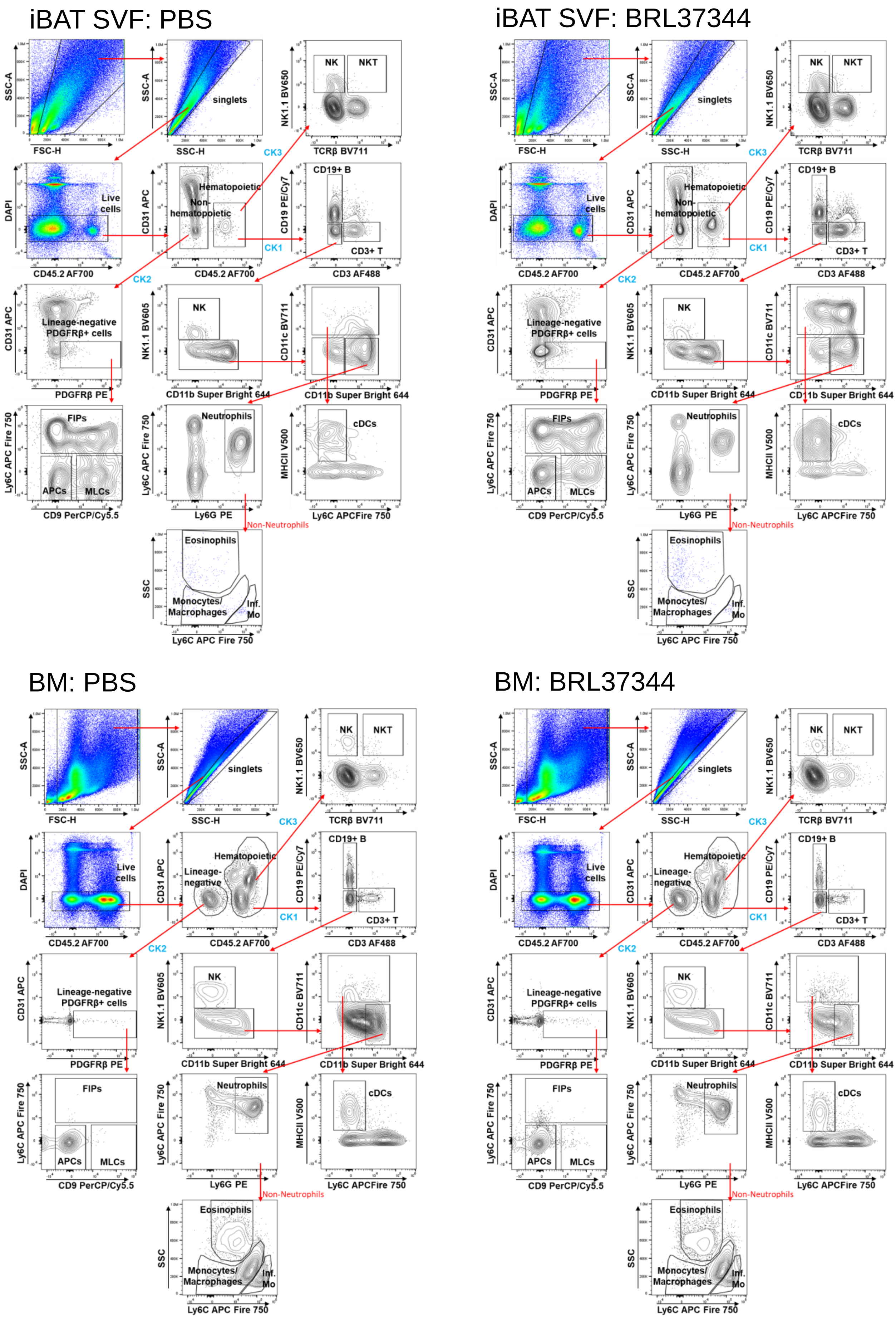

iBAT SVF: PBS
iBAT SVF: BRL37344
BM: PBS
BM: BRL37344
SSC-A
FSC-H
SSC-H
singlets
NK
NKT
NK1.1 BV650
TCRβ BV711
CK3
DAPI
Live cells
CD45.2 AF700
CD31 APC
Hematopoietic
Non-hematopoietic
Lineage-negative
CK1
CK2
CD19+ B
CD19 PE/Cy7
CD3+ T
CD3 AF488
Lineage-negative PDGFRβ+ cells
PDGFRβ PE
NK1.1 BV605
CD11b Super Bright 644
CD11c BV711
FIPs
APCs
MLCs
Ly6C APC Fire 750
CD9 PerCP/Cy5.5
Neutrophils
Ly6C APC Fire 750
Ly6G PE
cDCs
MHCII V500
Ly6C APCFire 750
Non-Neutrophils
Eosinophils
Monocytes/ Macrophages
Inf. Mo
SSC

Supplementary Figure 5. Gating strategy and representative flow cytometric plots of myeloid, lymphoid, and preadipocyte populations in iBAT SVF and femur bone marrow. Live hematopoietic cells were further characterized to identify five myeloid subpopulations with antibody cocktail 1 (CK1) (α-CD3, α-CD19 , α-Ly6C , α-Ly6G , α-CD11b , α-CD11c , and α-MHCII Abs). PDGFRβ+ progenitors were assessed with antibody cocktail 2 (CK2) (α-PDGFRβ, α-CD9 , and α-Ly6C Abs). NK and NKT cells populations were identified with antibody cocktail 3 (CK3) (α-NK1.1 and α-TCRβ Abs). NK: natural Killer cells, NKT: NK T cells, cDCs: Classical Dendritic Cells, Inf. Mo: Inflammatory Monocytes/Macrophages, APC: adipocyte precursor cells, FIP: fibro-inflammatory progenitors, MLC: mesothelial-like cells.

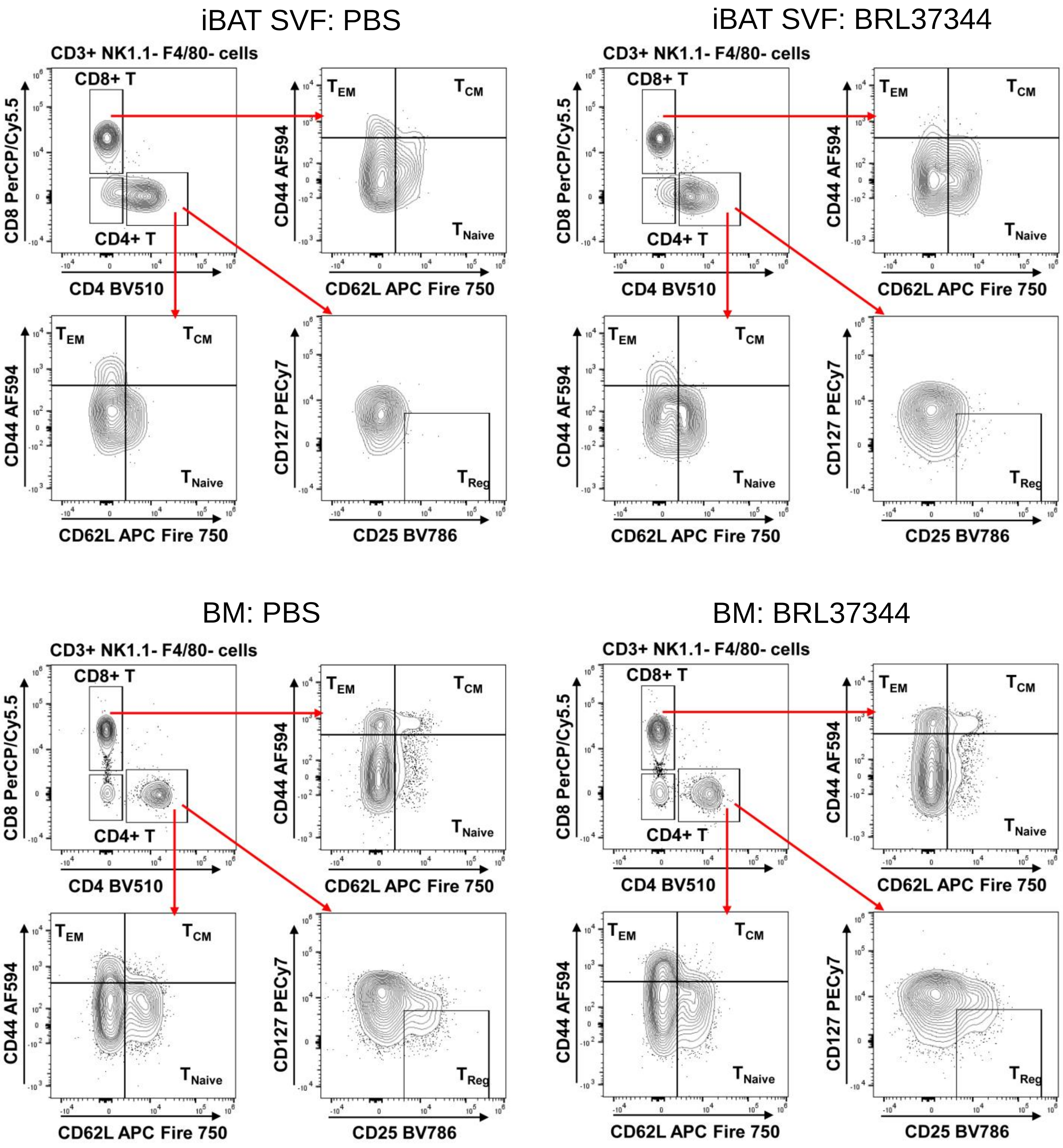

Supplementary Figure 6. Gating strategy and representative flow cytometric plots of T cell subsets in iBAT SVF and femur bone marrow. CD3+ NK1.1- F/480- live T cells were further characterized by CD4, CD8, CD62L, CD44, CD25, and CD127 expression. T_{EM}: Effector memory T, T_{CM}: Central memory T, T_{Naive}: Naïve T, T_{Reg}: Regulatory T.

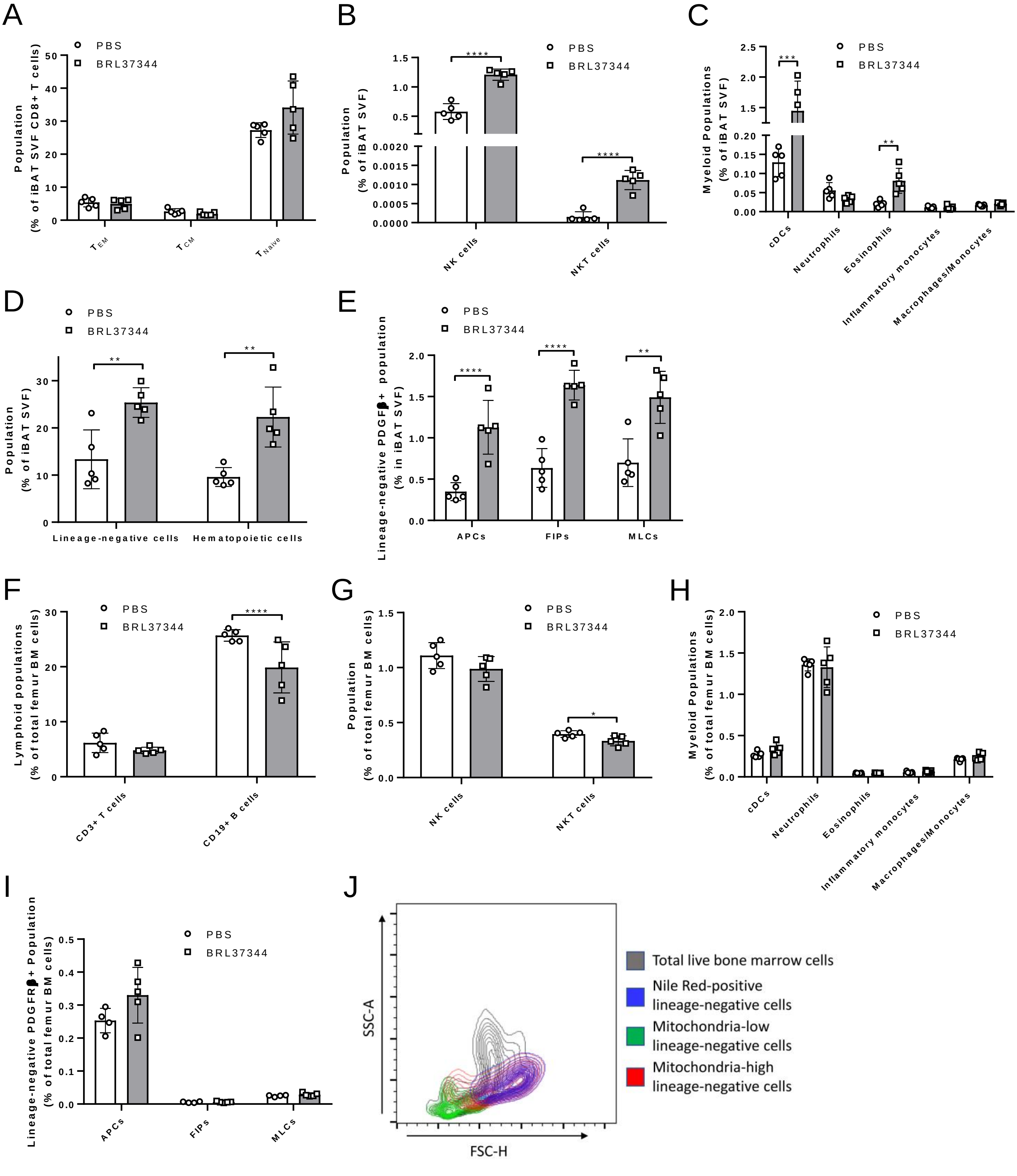

A
PBS
BRL37344
Population
(% of iBAT SVF CD8+ T cells)
T EM
T CM
T Naive
B
Population
(% of iBAT SVF)
NK cells
NKT cells
C
Myeloid Populations
(% of iBAT SVF)
cDCs
Neutrophils
Eosinophils
Inflammatory monocytes
Macrophages/Monocytes
D
Population
(% of iBAT SVF)
Lineage-negative cells
Hematopoietic cells
E
Lineage-negative PDGFRβ+ population
(% in iBAT SVF)
APCs
FIPs
MLCs
F
Lymphoid populations
(% of total femur BM cells)
CD3+ T cells
CD19+ B cells
G
Population
(% of total femur BM cells)
NK cells
NKT cells
H
Myeloid Populations
(% of total femur BM cells)
cDCs
Neutrophils
Eosinophils
Inflammatory monocytes
Macrophages/Monocytes
I
Lineage-negative PDGFRβ+ Population
(% of total femur BM cells)
APCs
FIPs
MLCs
J
SSC-A
FSC-H
Total live bone marrow cells
Nile Red-positive lineage-negative cells
Mitochondria-low lineage-negative cells
Mitochondria-high lineage-negative cells

Supplementary Figure 7. Flowcytometric analysis of iBAT and femur bone marrow cells. Frequencies of CD8+ T cell subsets (A), NK/NKT cells (B), myeloid subsets (C), lineage-negative cells (D), and preadipocytes (E) in iBAT SVF. Frequencies of lymphoid populations (F), NK/NKT cells (G), myeloid subsets (H) and preadipocytes (I) in femur bone marrow. Percentages of each population were assessed using FlowJo software and are graphed as mean ± SD (n = 5). Each spot represents an individual mouse. * $p < 0.05$; ** $p < 0.01$; *** $p < 0.001$; **** $p < 0.0001$. (J) FSC/SSC plot for femur bone marrow cells. Nile-Red-positive lineage-negative cells showed higher FSC/SSC values than mitochondria-low lineage-negative cells.

A

Animal ID #	WBC (K/ul)	Absolute Neutrophil cells (K/ul)	Absolute Lymphocyte cells (K/ul)	Absolute Monocyte cells (K/ul)	Absolute Eosinophil cells (K/ul)	Absolute Basophil cells (K/ul)	Neutrophil %	Lymphocyte %	Monocyte %	Eosinophil %	Basophil %	RBC (M/ul)	Hemoglobin (g/dL)	Hematocrit %	MCV (fL)	MCH (pg)	MCHC (g/dL)	RDW %	Platelets (K/uL)	MPV (fL)	Presence of clots
WT1	5.78	1.39	3.99	0.35	0.05	0.01	24.03	69.05	5.99	0.80	0.12	9.51	12.7	47.4	49.8	13.4	26.8	18.2	180	5.0	none
WT2	1.54	0.27	1.15	0.11	0.01	0.00	17.59	74.51	7.05	0.85	0.00	9.57	13.1	46.3	48.4	13.7	28.3	17.5	45	5.4	none
WT3	3.64	0.54	2.77	0.21	0.10	0.02	14.83	75.99	5.84	2.80	0.55	8.49	11.5	40.3	47.5	13.5	28.5	16.6	31	5.4	none
KO1	3.72	0.62	2.66	0.31	0.09	0.03	16.67	71.60	8.37	2.42	0.93	10.02	13.9	47.9	47.8	13.9	29.0	17.8	47	6.0	none
KO2	3.28	0.72	2.37	0.17	0.02	0.00	21.88	72.25	5.07	0.72	0.09	9.19	12.5	43.4	47.2	13.6	28.8	17.1	231	5.4	none
KO3	1.88	0.21	1.45	0.10	0.09	0.03	11.43	77.00	5.54	4.60	1.43	7.50	10.8	35.0	46.7	14.4	30.9	17.7	45	6.1	none
average WT	3.65	0.73	2.64	0.22	0.05	0.01	18.82	73.18	6.29	1.48	0.22	9.19	12.43	44.67	48.57	13.53	27.87	17.43	85.33	5.27	
average KO	2.96	0.52	2.16	0.19	0.07	0.02	16.66	73.62	6.33	2.58	0.82	8.90	12.40	42.10	47.23	13.97	29.57	17.53	107.67	5.83	
SD WT	2.12	0.58	1.42	0.12	0.05	0.01	4.72	3.66	0.66	1.14	0.29	0.61	0.83	3.82	1.16	0.15	0.93	0.80	82.28	0.23	
SD KO	0.96	0.27	0.63	0.11	0.04	0.02	5.23	2.95	1.79	1.94	0.68	1.28	1.55	6.55	0.55	0.40	1.16	0.38	106.81	0.38	
TTEST	0.63	0.59	0.62	0.76	0.72	0.44	0.62	0.88	0.98	0.45	0.24	0.74	0.98	0.59	0.15	0.16	0.12	0.85	0.79	0.09	

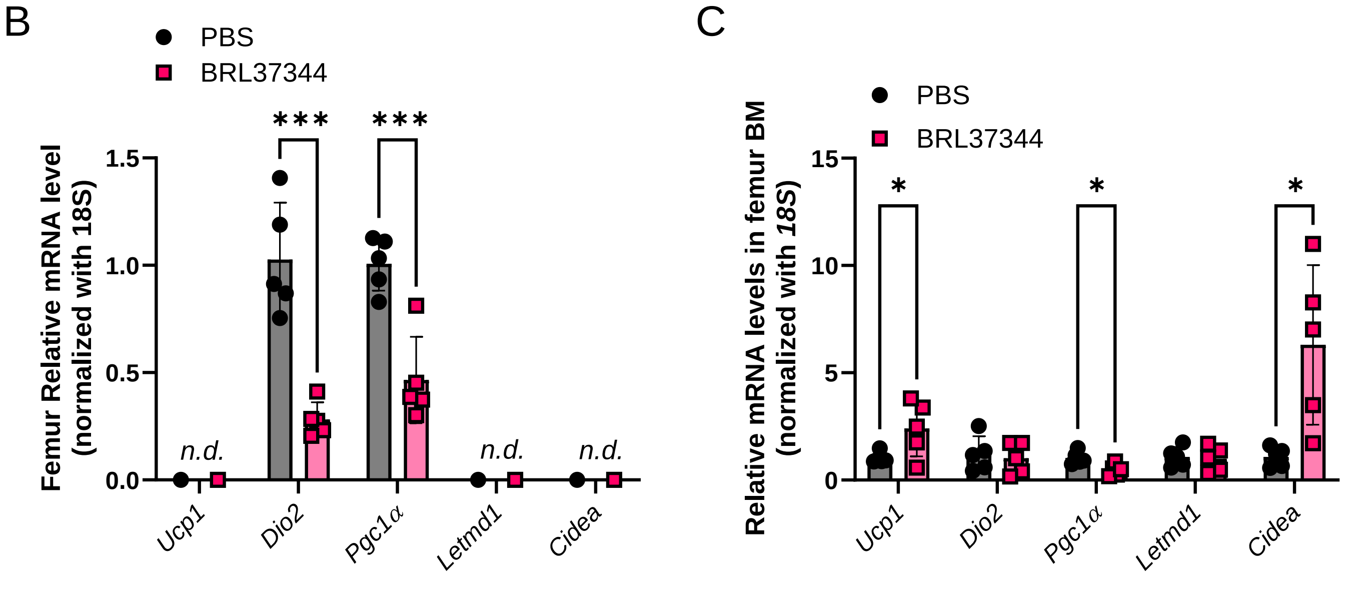

Supplementary Figure 8. (A) Analysis of CBC parameters in *Letmd1* KO and WT mice. There were no significant differences between groups. Relative mRNA expression of thermogenesis-related genes in femur bone marrow following 4d treatment of BRL37344 in *Letmd1* KO (B) and *Rag1* KO mice (C). Data are plotted as mean ± SD (n = 5). Each spot represents an individual mouse. * $p < 0.05$; *** $p < 0.001$.

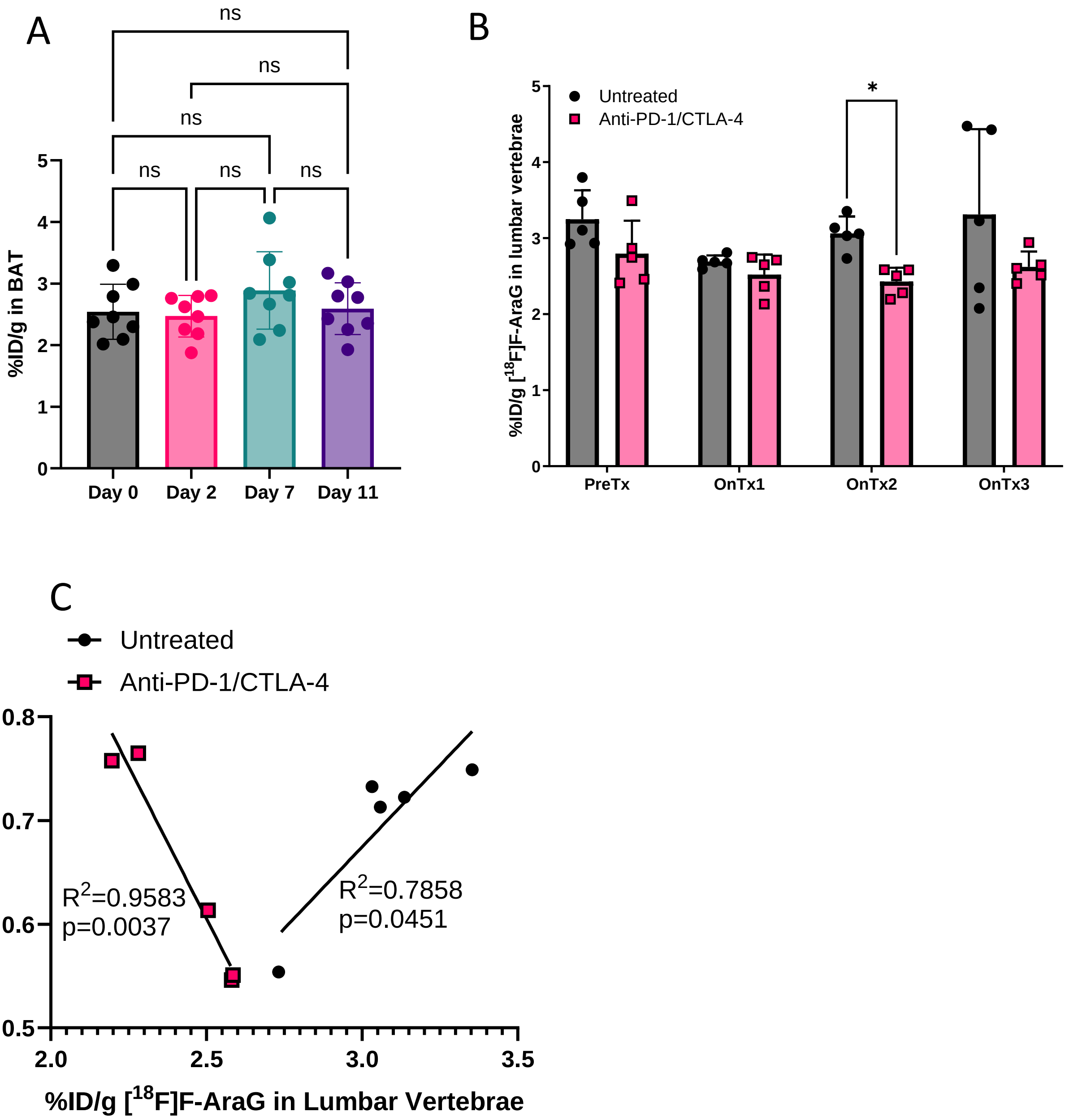

Supplementary Figure 9. (A) BAT activation is not observed in a subcutaneous MC38 tumor model. Mice were subcutaneously implanted with MC38 colon cancer cells and longitudinally imaged with [^{18}F]F-AraG when the tumors reached about 150 mm^3 in volume. The increase in BAT was not observed in MC38 bearing mice at any of the imaging time points. (B) After two checkpoint inhibitor injections, signal in the lumbar vertebrae was significantly different between treated and untreated mice. **C.** After two treatments, correlations between signal in the lumbar vertebrae and brain for treated and untreated mice were opposite in character. Untreated mice showed positive correlation, while in treated mice the correlation was negative. * $p \leq 0.05$; ** $p \leq 0.01$; *** $p \leq 0.001$; **** $p < 0.0001$.

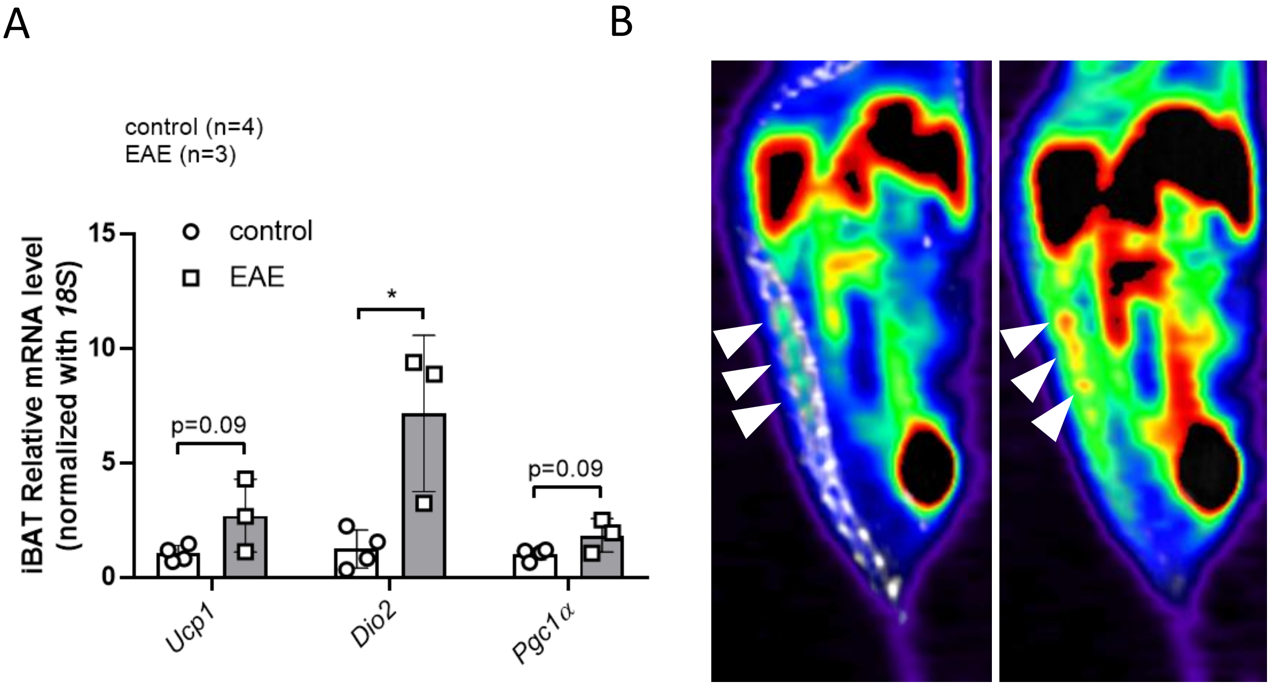

Supplementary Figure 10. (A) Relative mRNA expression of thermogenesis-related genes in iBAT of EAE mice. Expression of Dio2 was significantly increased in EAE mice, while expression of Ucp1 and Pgc1α showed a trend towards increase but did not reach statistical significance. Experiment performed at Washington University at St. Louis (protocol number 23-0323) (B) Expression[^{18}F]F-AraG accumulates in the vertebral bone marrow of CPZ-EAE mice. The uptake in the vertebrae shows a non-continuous, segmentary pattern, indicating uptake in the vertebral body (white arrows).

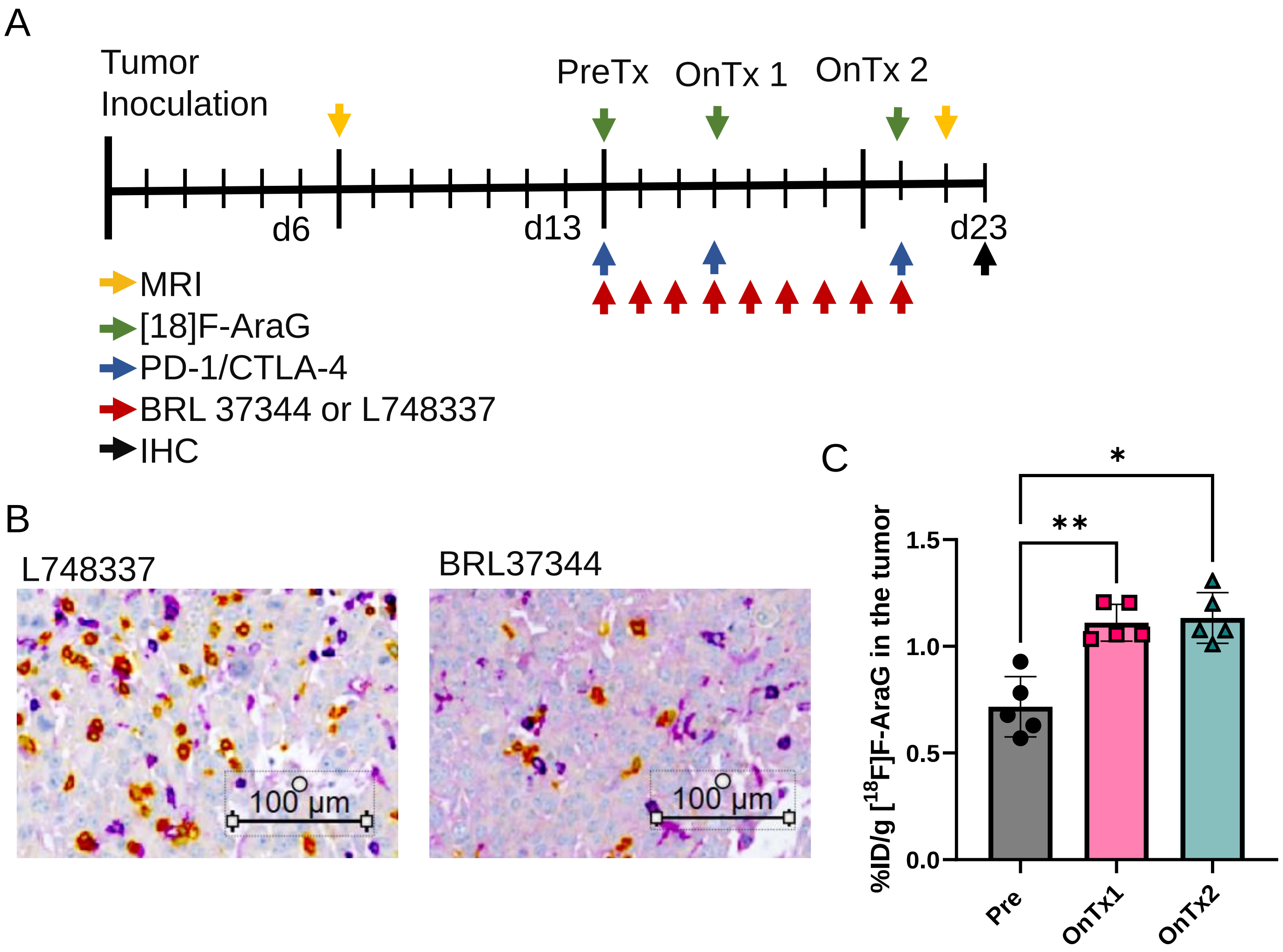

Supplementary Figure 11. Adrenergic signaling affects lymphocyte infiltration into the brain. (A) Mice carrying GBM tumors were longitudinally imaged during combinatorial therapy that consisted of checkpoint inhibitor antibodies and either adrenergic agonist (BRL37344, 10 mg/kg) or antagonist (L748337, 1 mg/kg). (B) Co-administration of adrenergic antagonist resulted in higher intratumoral immune infiltration. (C) An increase in brain tumor inflammation with check point inhibitor therapy combined with adrenergic antagonist was also evident by the increase in [^{18}F]F-AraG intratumoral signal. *P≤0.05, **P≤0.01

Supplementary Table 1. List of antibodies for flow cytometry

Antibodies	Source	Cat#
TruStain FcX™ PLUS (anti-mouse CD16/32)	BioLegend	156604
Alexa Fluor® 488 anti-mouse CD3ε	BioLegend	100321
Brilliant Violet 510™ anti-mouse CD4	BioLegend	100449
PerCP/Cyanine5.5 anti-mouse CD9	BioLegend	124818
Brilliant Violet 711™ anti-mouse CD11c Antibody	BioLegend	117349
PE/Cyanine7 anti-mouse CD19	BioLegend	152418
Brilliant Violet 785™ anti-mouse CD25	BioLegend	102051
APC anti-mouse CD31	BioLegend	102410
Alexa Fluor® 700 anti-mouse CD31	BioLegend	102444
Biotin anti-mouse CD31	BioLegend	102404
PE/Cyanine7 anti-mouse CD34	BioLegend	119326
Biotin anti-mouse/human CD44	BioLegend	103004
Biotin anti-mouse CD45.2	BioLegend	109804
Alexa Fluor® 700 anti-mouse CD45.2	BioLegend	109822
PE anti-mouse CD69	BioLegend	104508
PE/Cyanine7 anti-mouse CD127 (IL-7Rα)	BioLegend	135014
PE/Dazzle™ 594 anti-mouse F4/80	BioLegend	123146
Brilliant Violet 650™ anti-mouse Ly-6C	BioLegend	128049
PE anti-mouse Ly-6G	BioLegend	127607
Brilliant Violet 605™ anti-mouse NK-1.1	BioLegend	108753
Brilliant Violet 605™ anti-mouse CD279 (PD-1)	BioLegend	135220
PE anti-PDGFRb (CD140b)	BioLegend	136005
Brilliant Violet 711™ anti-mouse TCR β chain	BioLegend	109243
APC/Fire™ 750 Streptavidin	BioLegend	405250
Human TruStain FcX™	BioLegend	422302
V500 anti-Human CD8	BD Biosciences	560775
APC anti-human CD25	BioLegend	356110
PE anti-human CD279 (PD-1)	BioLegend	367404

Supplementary Table 2. List of qRT-PCR primers

gene	Forward	Reverse
Cidea	TGACATTCATGGGATTGCAGAC	GGCCAGTTGTGATGACTAAGAC
Dio2	GATGCTCCCAATTCCAGTGT	TGAACCAAAGTTGACCACCA
Letmd1	CTACCCACATTGCTGACGAC	GGGACAGCTCGGGTTCTT
Pgc1α	CCCTGCCATTGTTAAGACC	TGCTGCTGTTCCTGTTTTC
Ucp1	CACTCAGGATTGGCCTCTACG	GGGGTTTGATCCCATGCAGA
18S	AGTCCCTGCCCTTTGTACACA	CGATCCGAGGGCCTCACTA

Supplementary Table 3. Baseline patient demographics and PET metrics

**Subject**	**Age**	**Gender**	**BMI**	**Month of the Scan**	**Signal in the BAT left neck base/ right neck base (SUV max)**	**Signal in the confluence of sinuses (SUV max)**
1	65	Male	22	October	1.50/1.49	0.50
2	29	Female	22	March	2.32/1.86	0.55
3	31	Female	21	August	2.46/2.8	0.38
4	34	Female	25	October	1.72/1.87	0.62

Supplementary Table 4. [^{18}F]F-AraG uptake and cellular changes in BAT and BMAT after chronic adrenergic stimulation in different mouse models

	**Wild Type** (no deficiency in T cells or adipocytes)	**Letmd1-KO** (deficiency in adipocytes)	**Rag1-KO** (deficiency in T cells)
**[^{18}F]F-AraG signal in the BAT**	+	-	+
**[^{18}F]F-AraG signal in the BMAT**	+	-	-
**Cellular changes in BAT post adrenergic stimulation**	Increase in T cells Increase in brown adipocytes	Decreased mitochondrial content in iBAT adipocytes	No T cells
**Cellular changes in BMAT post adrenergic stimulation**	No increase in T cells, increase in mitochondria rich adipocytes)	Reduction of mitochondria-rich adipocyte population	No increase in mitochondrial content of adipocytes
